# Inhibition of translocator protein 18 kDa suppressed the progression of glioma via the ELAV-like RNA-binding protein 1/MAPK-activated protein kinase 3 axis

**DOI:** 10.1080/21655979.2022.2048992

**Published:** 2022-03-12

**Authors:** Jingya Wang, Peng Ren, Zhirui Zeng, Li Ma, Yunjun Li, Hongmei Zhang, Wenzhi Guo

**Affiliations:** a Department of Anesthesiology, The Seventh Medical Center of Chinese PLA General Hospital, Beijing, Dongcheng,China; b Department of Gastroenterology, The Affiliated Hospital of Weifang Medical University, Weifang, Shandong, China; c Guizhou Provincial Key Laboratory of Pathogenesis & Drug Research on Common Chronic Diseases, Department of Physiology, School of Basic Medical Sciences, Guizhou Medical University, Guiyang, Guizhou, China; d Department of Neurosurgery, The Seventh Medical Center of Chinese PLA General Hospital, Beijing, Dongcheng, China

**Keywords:** Glioma, TSPO, HUR, MAPKAPK3, proliferation, mobility

## Abstract

Glioma is the most common primary malignant brain tumors in adults. Despite considerable advances in treatment, the clinical outcome remains dismal. Translocator protein 18 kDa (TSPO), an evolutionarily conserved transmembrane protein, has always been found to be elevated in glioma, which predicts a poor prognosis. However, studies on the regulatory network of TSPO in glioma are limited. The Cancer Genome Atlas (TCGA) and our research group cohorts demonstrated that TSPO expression was also highly expressed in glioma tissues and glioma cell lines. Inhibition of TSPO expression significantly reduced glioma cell proliferation and mobility *in vitro*. Suppression of TSPO decreased the expression of MAPK-activated protein kinase 3 (MAPKAPK3) and increased the degradation rate of its mRNA. TSPO directly interacts with ELAV1-like RNA-binding protein 1 (HUR) and promotes the nuclear–cytoplasmic shuttling of HUR. Inhibition of HUR decreased MAPKAPK3 expression and cell proliferation and mobility, whereas overexpression of MAPKAPK3 reversed the effects. Overexpression of HUR in TSPO-knockdown cells enhanced the mRNA stability of MAPKAPK3. Furthermore, rescue experiments show that the HUR/MAPKAPK3 axis accounts for the TSPO-mediated effects on glioma cell proliferation and mobility. Together, our present study indicated that TSPO may promote the nuclear–cytoplasmic shuttling of HUR, thus increasing the mRNA stability of MAPKAPK3 and promoting the proliferation and mobility of glioma cells. The HUR/MAPKAPK3 axis may be key targets for blocking the effects of TSPO and may contribute to glioma therapy.

## GraphicalAbstract

**Figure:**
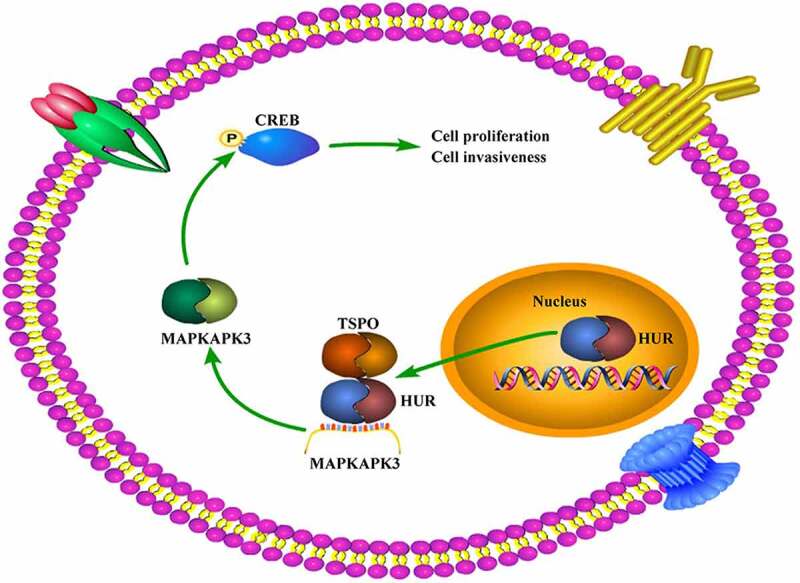

## Introduction

Glioma is the most fatal primary intracranial tumor in adults. Despite any extensive treatments, the treatment effect is not obvious, and the disease can easily relapse, leading to an average survival time shorter than 1.5 years [1,2]. Numerous disease features, such as being heterogeneous, aggressive invasion, and resistance to many anticancer treatments, contribute to the challenge of glioma treatment [3]. One important factor is the difficulty to effectively activate intracellular apoptosis pathways [4]. Given the current research status, deep understanding of the mechanisms underlying glioma will facilitate its diagnosis and treatment.

The translocator protein 18 kDa (TSPO), previously known as the peripheral benzodiazepine receptor, is an evolutionarily conserved 18 kDa transmembrane protein that always forms a protein complex composed with VDAC1 and ANT that reside in both the outer and inner mitochondrial membranes, which facilitates the transport of cholesterol from the outer to the inner mitochondrial membrane, thus synthesizing steroids and neurosteroids [5–7]. Not least due to its location on the outer mitochondrial membrane, TSPO is essential in a broad spectrum of biological functions and has recently received increasing attention as well as extensive investigation as a specific biomarker in central nervous system diseases including glioma [8]. In glioma, an increasing body of scientific evidence have been accumulated to prove that TSPO expression levels are markedly elevated, hinting at the considerable role of TSPO in facilitating tumorigenesis and progression in glioma [9]. TSPO appears to be part of the mitochondria-to-nucleus signaling pathway, regulating nuclear gene expression in glioma cells [10]. Furthermore, TSPO ligands or inhibitors have also been considered and designed as potential drugs for cancer treatment [11]. However, the regulatory network of TSPO is still largely unknown.

HUR, a member of the ELAV gene family RNA-binding protein, is an important mRNA stabilizer described to date [12]. HUR is primarily localized in the nucleus and is capable of nuclear–cytoplasmic shuttling in response to the cellular environmental stress of a variety of signals [13]. Previous research reported that the HUR protein is overexpressed in glioma, necessary for tumor growth, and serves as a positive regulator of tumor-promoting genes in glioma [14]. Moreover, HUR regulates the expression of the mitogen-activated protein kinase (MAPK) pathway via mRNA stability in glioma [15,16].

MAPK cascades are key signaling pathways involved in a wide variety of cellular processes, such as proliferation, differentiation, apoptosis, and stress responses, through the activation of the MAPK-activated protein kinase 3 (MAPKAPK3) [17,18]. It is reported that approximately 88% of gliomas occur along with MAPK pathway alteration, as MAPK genes determine the invasive or proliferative phenotypes [19,20]. cAMP-response element-binding protein (CREB) is an oncoprotein that lies downstream of the MAPK pathways and regulates cell cycle factor expression in glioma cells [21].

This study aimed to determine the novel molecular mechanism of TSPO in glioma. It is demonstrated that TSPO recruits HUR shuttling from the nucleus to cytoplasm and enhances the stability of the MAPKAPK3 mRNA, activating the oncogene CREB, thus promoting glioma cell proliferation and mobility. Our findings underscore that the HUR/MAPKAPK3 axis is a key downstream of TSPO, and targeting this axis may be a potential strategy for blocking the effects of TSPO, contributing to glioma therapy.

## Materials and methods

### Bioinformatics analysis in The Cancer Genome Atlas (TCGA)

The expression and relationship between patient prognosis of target genes in glioma including low-grade glioma (LGG) and glioblastoma (GBM) in the samples of The Cancer Genome Atlas (TCGA) database were analyzed using the online tool GEPIA (http://gepia.cancer-pku.cn/). |Log Change| > 1 and *P* < 0.01 were set as thresholds to consider significant differences in the expression between glioma tissues and adjacent tissues. *P* < 0.05 was set as the cutoff to consider significant relationships between target genes and patient prognosis.

### Glioma tissue collection

A total of 47 patients, aged 40–70 years, consented to participate in the current study. All patients’ sections were obtained from the Department of Pathology, The Seventh Medical Center of Chinese PLA General Hospital (Beijing, China) between June 2017 and May 2020. All samples were diagnosed as primary glioma by three pathologists independently, according to the WHO Classification of Tumors of the Central Nervous System. None of the patients received radiotherapy and chemotherapy before sample collection. All patients signed the written informed consent, and the Human Ethics Committee of our institution (approval number: 2020–106) approved the tissue collection and use.

### Immunohistochemistry and scoring

All glioma specimens were fixed with 4% paraformaldehyde (cat no. 30,525–89-4, Sigma-Aldrich, USA), embedded in paraffin, and cut into 4-μm-thick slides. Briefly, slides were heated and dehydrated in xylene and graded alcohols. After antigen retrieval with 0.01 M citrate (Servicebio, Wuhan, China) buffer at pH 6.0 at 95°C for 20 min, the endogenous peroxidases in the tissue slides were blocked using 3% hydrogen peroxide, and then 5% bovine serum albumin (Wuhan Boster Biological Technology, Ltd., China) was added to block nonspecific binding. The slides were incubated as the TSPO (cat no. ab109497; Abcam, USA) primary antibodies at 4°C overnight. Then, sections were washed with PBS and incubated with horseradish peroxidase-conjugated secondary antibodies (Servicebio, Wuhan, China) for 2 h. Diaminobenzidine and hematoxylin (ZSGB-BIO, Beijing, China) were used for color development, and images were obtained using an orthophoto microscope (BX53 version; Olympus, Japan). Target protein levels were calculated based on the intensity scores and the concrete method described in our previous publication [2].

### Cell culture and transient transfection

Normal human astrocytes (NHAs) were purchased from the national model and characteristic experimental cell resource library (Shanghai, China), and the three glioma cells including U87, U251, and T98G were obtained from the American Type Culture Collection (USA). All cells were cultured in DMEM (Invitrogen, California, USA) containing 10% fetal bovine serum (FBS; Invitrogen) at 37°C with 5% CO_2_. TSPO small interfering RNA (siRNA; si-TSPO), HUR siRNA (si-HUR), negative control siRNA (NC) were obtained from GeneCopoeia (Guangzhou, China). The si-TSPO sequence was 5’-UCUUUGGUGCCCGACAAAUTT-3’, the si-HUR sequence was 5’-AAGAGGCAAUUACCAGUUUCA-3’, and the NC sequence was 5’-UUCUCCGAACGUGUCACGUTT-3’. The plasmids overexpressing MAPKAPK3 and HUR were obtained from Juneng Huida Biological Co., Ltd. (Wuhan, China). For transient transfection, cells were grown on 6-well plates in 50% confluence and transfected with siRNAs and plasmids using Lipofectamine 2000 (Invitrogen).

### Western blotting

The total proteins in glioma cells were extracted using a radioimmunoassay precipitation lysis buffer (Wuhan Boster Biological Technology, Ltd.) containing phenylmethylsulfonyl fluoride (Guangzhou Dongrui Technology Co., Ltd., Guangzhou, China). The protein concentrations of the samples were determined using a bicinchoninic acid method (Wuhan Boster Biological Technology, Ltd.). The 10% sodium dodecyl sulfate polyacrylamide gels (Beyotime Biotechnology, Suzhou, China) were employed in separating proteins. Then, the separating proteins were transferred to polyvinylidene fluoride membranes (Thermo Fisher Scientific, Waltham, MA, USA). After blocking the membranes with skimmed milk powder (Beyotime Biotechnology), they were incubated with primary antibodies, containing TSPO (1:1000, cat no. ab109497; Abcam), E-cadherin (1:1000, cat no. 20,874-1-AP; Proteintech Group, Wuhan, China), N-cadherin (1:1000, cat no. 22,018-1-AP; Proteintech Group), vimentin (1:1000, cat no. 10,366-1-AP; Proteintech Group), β-catenin (1:1000, cat no. 51,067-2-AP; Proteintech Group), Slug (1:1000, cat no. 12,129-1-AP; Proteintech Group), MAPKAPK3 (1:1000, cat no. 15,424-1-AP; Proteintech Group), p-MAPKAPK3 (1:250, cat no. SPC-1016; StressMarq Biosciences Inc., USA), CREB (1:1000, cat no. 12,208-1-AP; Proteintech Group), p-CREB (1:1000, cat no. #9198; Cell Signaling Technology, USA), HUR (1:1000, cat no. 11,910-1-AP; Proteintech Group), H3 (1:1000, cat no. 17,168-1-AP; Proteintech Group), and α-tubulin (1:2000, cat no. 11,224-1-AP; Proteintech Group), for 12 h at 4°C. Washing twice with Tris‐buffered saline containing 0.1% Tween‐20, the membranes were incubated with the secondary antibody and visualized using an enhanced chemiluminescent reagent. α-tubulin was used as the loading control to calculate the relative expression of total protein and plasmosin; H3 was set as the loading control to calculate the relative expression of nuclear protein.

### Cell count kit-8

The Cell Counting Kit-8 (CCK-8; Dojindo Molecular Technologies Inc., Japan) was used to detect the ability of glioma cells. Briefly, a total of 3000 glioma cells were set per well in a 96-well plate (n = 6 for per group) and cultured at 37°C for 24 and 48 h. Then, after changing the medium, 10 μL CCK-8 was added and cultured for 2 h. The absorbance of each well was detected at 450 nm wavelength using a multimode reader (Bio-Rad Laboratories, USA). This experiment perform triplicates.

### 5-Ethynyl-2-deoxyuridine (EDU) incorporation assay

The 5-ethynyl-2-deoxyuridine (EDU) assay was performed according to the manufacturer specification of BeyoClick™ EDU-488 cell proliferation detection kit (Beyotime Biotechnology, Hangzhou, China). Briefly, glioma cells cultured in a confocal dish, after transfection, and an EDU reagent were added and incubated for 2 h. After washing with phosphate-buffered saline (PBS) twice, the cells were fixed with 4% paraformaldehyde for 30 min. Then, the cells were incubated with Apollo staining reaction liquid for 30 min to detect the positive cells. Following staining with 4,6-diamino-2-phenylindole to identify the nucleus, immunofluorescence was observed through a fluorescence microscope at 488 nm.

### Wound healing assay

Cells were cultured in serum-free medium for 24 h and then treated with mitomycin (1 µg/mL) to inhibit cell division. Then, a 200-µL pipette tip was employed to scrape a wound in monolayer, while the floating cells were washed with PBS for three times. Serum-free DMEM was used to culture cells, and the wound healing condition was recorded from 0 to 24 h. The ability of cells to migrate was measured through the area of scratch healing.

### Transwell assay

Glioma cells (5 × 10^4^ cells/well) were resuspended in 200 μL DMEM without FBS and seeded onto the upper transwell chambers (Invitrogen) precoated with a Matrigel (Invitrogen), while a 700 μL medium containing 10% FBS was added into the lower transwell chambers. After 24 h, the invading cells were fixed and stained with 0.5% crystal violet. Finally, five random fields were photographed using an inverted microscope, and the average invasive number of cells in per field was counted.

### Immunofluorescence and confocal microscopy

Glioma cells were seeded into confocal dishes, immobilized with 4% formaldehyde, and permeabilized with 0.2% Triton X (Beyotime Biotechnology, Suzhou, China). Following blocking with 5% BSA, the cells were stained with the anti-E-cadherin (1:200), Slug (1:200), β-catenin (1:200), TSPO (1:200), MAPKAPK3 (1:200), and HUR (1:200) antibodies overnight at 4°C. After washing with PBS and incubating with CY3 or FITC-labeled second antibody, the cell nucleus was stained with a 4,6-diamino-2-phenylindole reagent and visualized using a Ti2-U fluorescence microscope (Nikon, Japan) or Axiotron 300 XYZ confocal microscopy (Zeiss, Germany).

### Quantitative real-time PCR

Total RNA was isolated from cultured cells using the TRIzol reagent, and complementary DNAs (cDNAs) were synthesized with 2 μg total RNA using the RevertAid First Strand cDNA Synthesis Kit (Thermo Fisher Scientific). RT-qPCR was performed to detect variation of specific gene expression using Aceq Universal SYBR qPCR Master Mix (Vazyme, Nanjing, China) on a Roche LightCycler 480 System (Roche, Shanghai, China). α-tubulin was used as a loading control. The 2^−ΔΔ^Ct method was used to measure the relative expression of target genes. The primers used in this study were as follows:

TSPO forward: 5’-CCTGCTCTACCCCTACCTGG-3’

TSPO reverse: 5’-GCCATACGCAGTAGTTGAGTG-3’

MAPKAPK3 forward: 5’- GGAAGGTGGTGAGTTGTTCAG-3’

MAPKAPK3 reverse: 5’-GCCAATATCCCGCATTATCTCTG-3’

α-tubulin forward: 5’-TCGATATTGAGCGTCCAACCT-3’

α-tubulin reverse: 5’-CAAAGGCACGTTTGGCATACA-3’

### Immunoprecipitation (IP) and mass spectrum identification

Briefly, a weak radioimmunoassay precipitation lysis buffer (Beyotime Biotechnology, Hangzhou, China) was used to lyse U251 cells. The cell lysates were then centrifuged at 12,000 g for 20 min at 4°C, and then, supernatants were incubated with TSPO antibodies overnight at 4°C, followed by incubation with protein A/G‐coated agarose beads (Merck, USA) for another 4 h at 4°C. Subsequently, samples were washed with cold immunoprecipitation (IP) buffer for three times, and the supernatants were removed through centrifugation at 2000 g for 1 min. The proteins were then separated from the beads using a loading buffer for 5 min at 95°C. The supernatants were collected and detected with the indicated antibodies through blotting and mass spectrometry.

### Statistical analysis

SPSS version 19.0 was used for all statistical analyses. Student’s t-test and one-way analysis of variance combined with least significant difference t-test were adopted to analyze differences between two groups and multiple groups, respectively. *P*-values <0.05 were considered to be statistically significant.

## Results

It was demonstrated that TSPO was highly expressed in glioma tissues and cell lines. Inhibition of TSPO significantly decreased the proliferation and mobility of U251 and U87 cells, whereas it had no obvious effects on NHA cells. MAPKAPK3 was co-expressed with TSPO in glioma tissues, whereas suppression of TSPO inhibited the expression of MAPKAPK3 in U251 and U87 cells and increased its mRNA degradation. TSPO directly binds with HUR and promotes its shuttling from the nucleus to the cytoplasm in U87 and U251 cells. Knockdown of HUR inhibited the expression of MAPKAPK3 and the proliferation and mobility of U251 and U87 cells, whereas overexpression of MAPKAPK3 can reverse these effects. Similarly, overexpression of HUR in TSPO-knockdown cells can obviously rescue the mRNA degradation of MAPKAPK3. Overexpression of HUR and MAPKAPK3 both significantly relieved the suppressive effects of TSPO knockdown on the proliferation and mobility of U251 and U87 cells.

### Elevated expression of TSPO was observed in glioma tissues and cell lines

**Figure 1.**
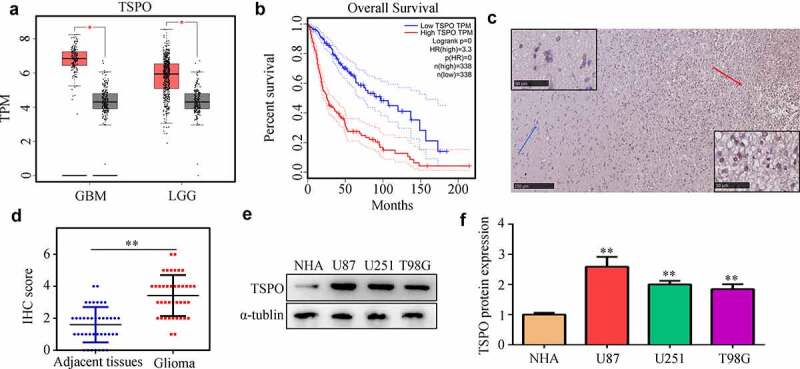
**TSPO expression was increased in glioma**. (a) Expression of TSPO in glioma and adjacent normal tissues in TCGA database. (b) Increased expression of TSPO in glioma tissues predicted poor outcome in TCGA database. (c-d) Expression of TSPO in glioma and adjacent tissues from our research cohort. (e-f) Protein expression level of TSPO in NHA, U251, U87, and T98G cells. ** *P* < 0.01.

According to data from TCGA, TSPO was highly expressed in glioma tissues (Figure 1a), whereas increased expression of TSPO was associated with poor prognosis (Figure 1b). Similarly, in the glioma tissues from our research cohort, TSPO was also highly expressed compared with that of adjacent tissues (Figure 1c–d). Furthermore, we found that TSPO expression was higher in U251, U87, and T98 cells compared with that of NHA cells (Figure 1e–f).

### Knockdown of TSPO inhibited the proliferation and mobility of glioma

**Figure 2.**
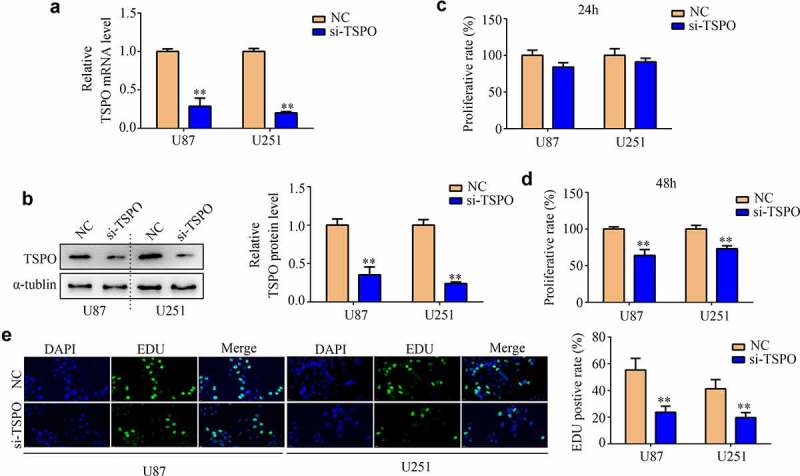
**Inhibition of TSPO suppressed the proliferation of U251 and U87 cells**. (a) Transfected efficiency of targeting TSPO siRNA of U251 and U87 cells was detected through qRT-PCR. (b) Transfected efficiency of targeting TSPO siRNA of U251 and U87 cells was detected through Western blotting. (c) CCK-8 assay was used to detect the proliferation of U87 and U251 cells with TSPO inhibition in 24 h. (d) CCK-8 assay was used to detect the proliferation of U87 and U251 cells with TSPO inhibition in 48 h. (e) The EDU assay showed that decreased TSPO expression inhibited the proliferation of U87 and U251 cells. ** *P* < 0.01.

**Figure 3.**
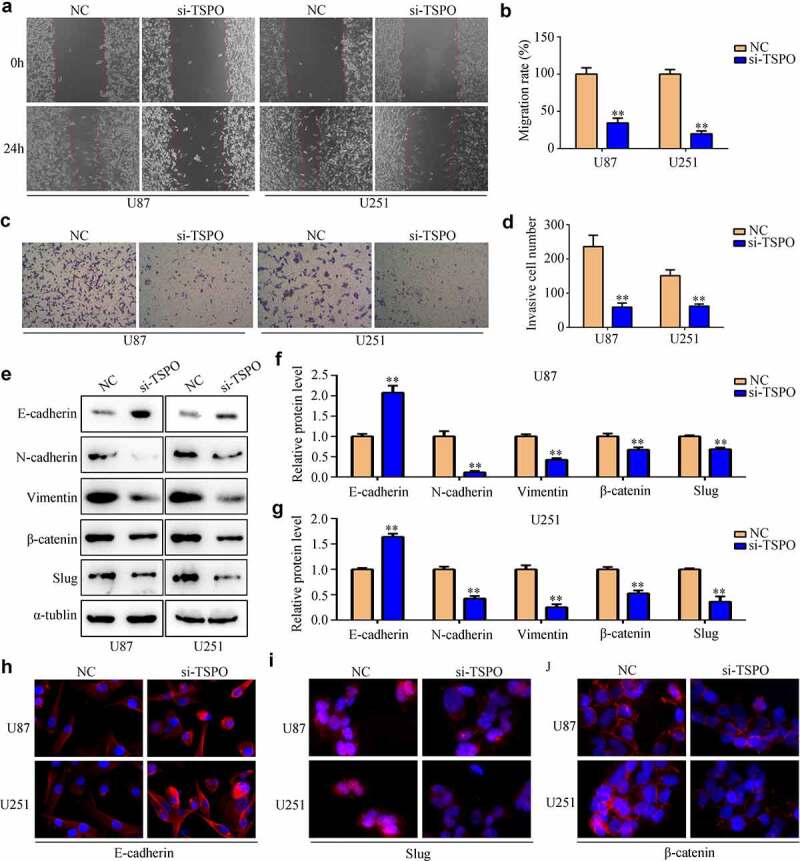
**Inhibition of TSPO decreased the migration and invasion of U251 and U87 cells**. (a–b) Wound healing assays were used to detect the wound healing rate of U251 and U87 cells with TSPO knockdown. The wound healing rate represented migration ability. (c-d) Transwell invasion assays were used to evaluate the invasive cell number in each field of the NC and si-TSPO groups. (e–g) Western blotting was used to detect EMT biomarkers including E-cadherin, N-cadherin, vimentin, β-catenin, and Slug in the NC and si-TSPO groups. (h-j) Immunofluorescence was used to detect the expression of E-cadherin, Slug, and β-catenin in U87 and U251 cells with TSPO suppression. ** *P* < 0.01.

Glioma cell lines U87 and U251 were transfected with NC and TSPO siRNA. Both qRT-PCR and Western blotting results indicated that TSPO siRNA significantly reduced the expression of U87 and U251 cells (Figure 2a-b). CCK-8 assays indicated that reduced TSPO expression inhibits the cell viability of U87 and U251 cells in 48 h (Figure 2c-d). Moreover, EDU assays indicated that reduced TSPO expression inhibits the rapid proliferation of U87 and U251 cells (Figure 2e). Similarly, U87 and U251 cells with lower TSPO expression exhibited decreased migration (Figure 3a-b) and invasion abilities (Figure 3c-d). Previous studies revealed that epithelial to mesenchymal transition (EMT) is a key process during mobility in numerous types of cancer [22]. Then, the expression levels of several EMT biomarkers in U87 and U251 cells were determined. Western blotting results indicated that E-cadherin expression levels were upregulated in U87 and U251 cells with low TSPO expression level, whereas N-cadherin, vimentin, β-catenin, and Slug expressions were decreased (Figure 3e-g). Similar to the results of Western blotting, immunofluorescence results also demonstrated that E-cadherin expression was markedly elevated in U87 and U251 cells, whereas TSPO was inhibited (Figure 3h), and Slug and β-catenin were decreased (Figure 3i–j). Interestingly, while the expression of TSPO was decreased in NHA cells (**Supplementary Fig. 1A–C**), a significant change in the proliferation (**Supplementary Fig. 1D**) and mobility (**Supplementary Fig. 1E**) of NHA was not found. These results indicated that TSPO may act as an oncoprotein to promote the proliferation and mobility of glioma cells.

### Inhibition of TSPO expression decreased the mRNA stability of MAPKAPK3

**Figure 4.**
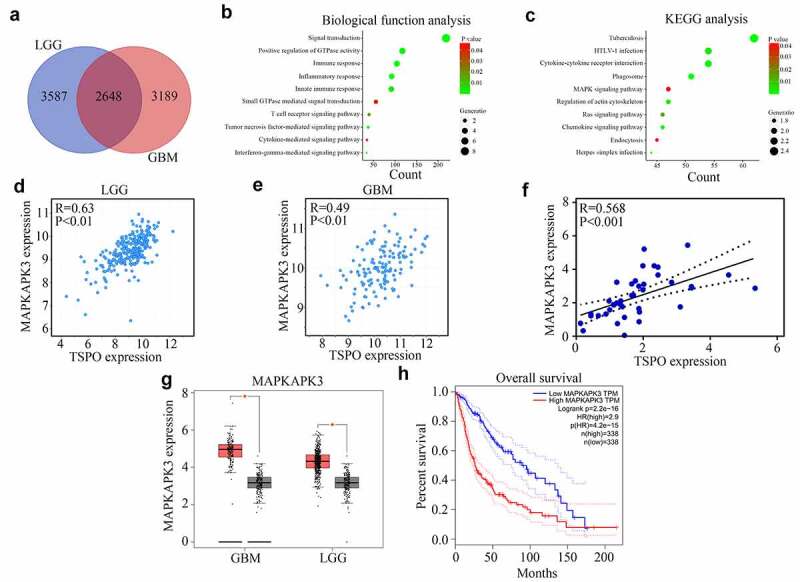
**MAPKAPK3 was significantly co-expressed with TSPO**. (a) Co-expression genes of TSPO in glioma (including LGG and GBM) according to data from TCGA database were identified. (b-c) Biological function and KEGG analysis of TSPO co-expression genes were performed. (d-e) Correlation between TSPO and MAPKAPK3 expression was identified from LGG and GBM tissue samples, respectively, in TCGA database. (f) Correlation between TSPO and MAPKAPK3 expression was identified from glioma tissues in our cohort. (g–h) The expression of MAPKAPK3 in patients with glioma and its relationship with survival rates are shown according to TCGA database.

**Figure 5.**
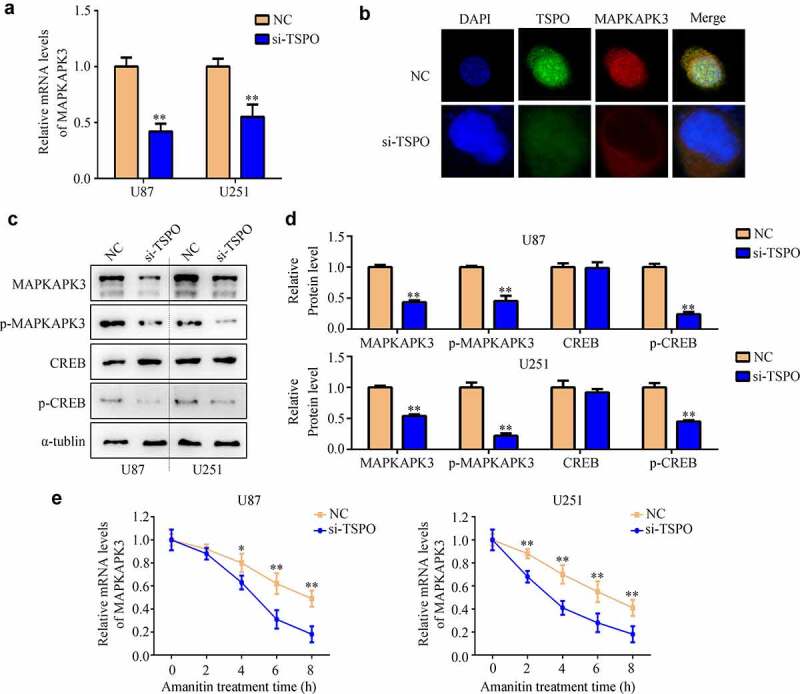
**Inhibition of TSPO decreased the expression of MAPKAPK3 and increased its mRNA degradation**. (a) qRT-PCR was used to detected the MAPKAPK3 mRNA levels in U87 and U251 cells with TSPO inhibition. (b) Co-immunostaining in U251 cells indicated that cells with TSPO knockdown had a lower MAPKAPK3 signal. (c-d) Western blotting showing the protein level of MAPKAPK3, p-MAPKAPK3, and p-CREB was decreased under TSPO inhibition. (e) Amanitin (0.1 µM) was used to inhibit mRNA synthesis at 0, 2, 4, 6, and 8 h, and qRT-PCR results indicated that inhibition of TSPO expression increased the degradation rate of the MAPKAPK3 mRNA. * *P* < 0.05; ** *P* < 0.01.

To further explore the molecular mechanism of TSPO in glioma, first, the co-expression genes of TSPO in glioma were analyzed according to the data from TCGA database. A total of 2648 genes were found (Figure 4a). Biological process enrichment and KEGG analyses showed that TSPO co-expressed genes were enriched in ‘signal transduction,’ ‘positive regulation of GTPase activity,’ ‘immune response,’ ‘inflammatory response,’ ‘innate immune response,’ ‘small GTPase-mediated signal transduction,’ ‘MAPK signaling pathway,’ and ‘Ras signaling pathway’ (Figure 4b–c). It was widely known that the MAPK signaling pathway was involved in glioma development [20]. Close attention must be paid to the MAPK signaling pathway, in which a member of this pathway, named MAPKAPK3, was significantly co-expressed with TSPO in both TCGA glioma tissues and our glioma tissues (Figure 4d-f). We found that MAPKAPK3 was highly expressed in glioma tissues and high MAPKAPK3 expression was associated with poor prognosis (Figure 4g-h). Moreover, we found that inhibition of TSPO expression significantly decreased the mRNA level of MAPKAPK3 (Figure 5a). Through co-immunostaining with TSPO and MAPKAPK3 in U251 cells, we found that cells with low TSPO expression had a weaker MAPKAPK3 signal (Figure 5b). Moreover, through Western blotting, it was found that both MAPKAPK3 and p-MAPKAPK3 were reduced in U251 and U87 cells with TSPO knockdown (Figure 5c-d). As CREB is a downstream target of MAPKAPK3, the expression of CREB was detected. A significant change in the total expression of CREB was not found, whereas p-CREB was significantly decreased in U251 and U87 cells with TSPO inhibition (Figure 5c-d). Furthermore, while amanitin (0.1 µM) was used to inhibit mRNA synthesis, inhibition of TSPO expression increased the degradation rate of the MAPKAPK3 mRNA (Figure 5e).

### TSPO recruited HUR from the cell nucleus to the cytoplasm

**Figure 6.**
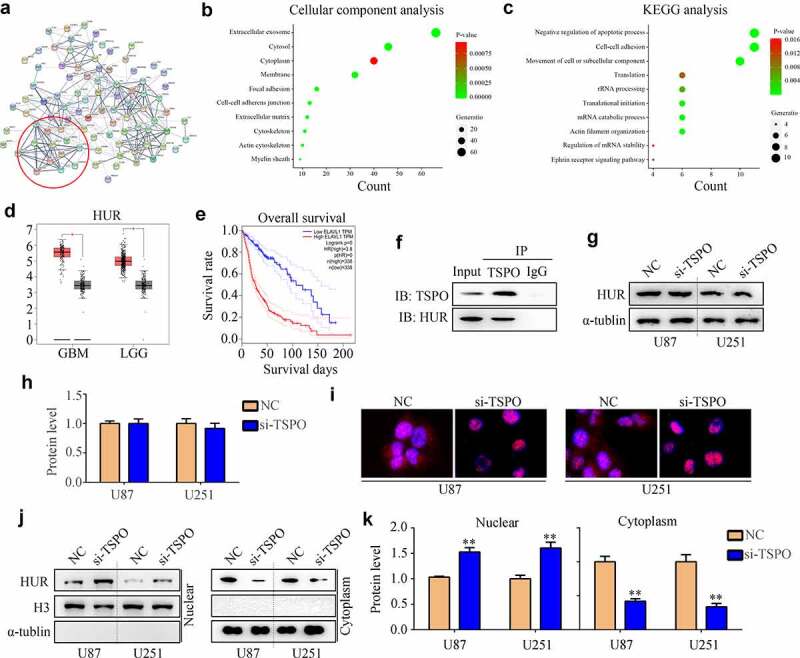
**TSPO recruited HUR from the nuclear to the cytoplasm**. (a) Mass spectrometry was used to identify the interacting proteins of TSPO. (b-c) Cellular composition enrichment and KEGG analysis of TSPO interacting proteins were performed. (d-e) The expression of HUR in patients with glioma and its relationship with survival rates were shown according to TCGA database. (f) Western blotting indicated the direct binding of HUR with TSPO. (g–h) Western blotting indicated that inhibition of the TSPO did not change the protein expression of HUR. (i) Immunofluorescence indicated that HUR shuttling from the nucleus to the cytoplasm in U87 and U251 cells was inhibited under TSPO inhibition. (j–k) Nuclear and cytoplasmic fractions were extracted, and Western blotting was used to detect the expression of HUR in nuclear and cytoplasmic fractions in U251 and U87 cells. ** *P* < 0.01.

To explore how TSPO regulated the mRNA stability of MAPKAPK3, mass spectrometry was used to identify the interacting proteins of TSPO (Figure 6a). Interestingly, cellular composition enrichment analysis showed that interacting proteins of TSPO was enriched in the cytoplasm (Figure 6b). Biological process enrichment analysis showed that interacting proteins of TSPO was enriched in the mRNA catabolic process and regulation of mRNA stability (Figure 6c). HUR (also named ELAVL1), an enriched protein, was highly expressed in glioma and negatively associated with patient prognosis (Figure 6d-e). Similarly, the IP assay indicated the HUR directly binds with TSPO (Figure 6f). Suppression of TSPO expression did not change the expression of HUR (Figure 6g-h), but recruited HUR shuttling from the nucleus to the cytoplasm (Figure 6i-k).

### HUR increased the mRNA stability of MAPKAPK3 and regulated cell proliferation and mobility

**Figure 7.**
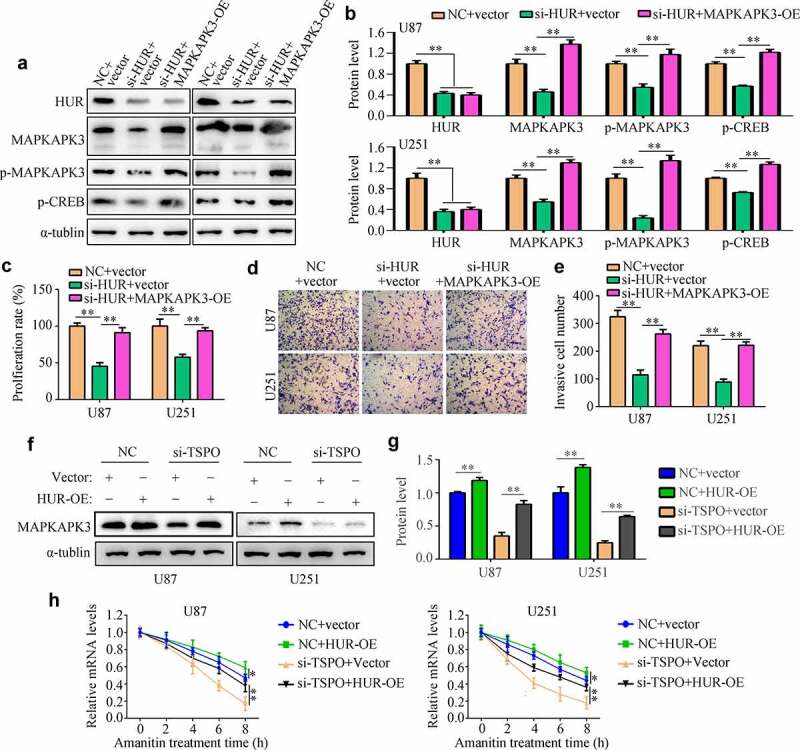
**HUR regulated MAPKAPK3 expression and cell proliferation and mobility**. U251 and U87 cells were treated with the NC siRNA + vector, HUR siRNA + vector, and HUR siRNA + MAPKAPK3 overexpression plasmid (MAPKAPK3-OE). (a–b) Western blotting was used to detect the protein expression of HUR, MAPKAPK3, p-MAPKAPK3, and p-CREB in each group. (c) The CCK-8 assay was used to detect the cell viability in each group in 48 h. (d-e) Transwell assays were used to detect the invasive cell numbers in each field in each group. (f–g) Western blotting was used to detect the expression of MAPKAPK3 in TSPO knockdown cells with HUR overexpression. (h) Amanitin (0.1 µM) was used to inhibit the mRNA synthesis for 0, 2, 4, 6, and 8 h, and qRT-PCR results indicated that overexpression of HUR increased the mRNA stability of MAPKAPK3 in U87 and U251 cells with TSPO inhibition. * *P* < 0.05; ** *P* < 0.01.

Through Western blotting, inhibition of HUR obviously reduced the expression of MAPKAPK3, p-MAPKAPK3, and p-CREB, whereas overexpression of the MAPKAPK3 reversed the effects of HUR (Figure 7a-b). HUR inhibition significantly reduced the viability of U251 and U87 cells in 48 h, whereas overexpression of MAPKAPK3 significantly relieved its effects (Figure 7c). Similarly, suppression of HUR significantly reduced the invasiveness of U251 and U87 cells in 24 h, whereas overexpression of MAPKAPK3 significantly relieved its effects (Figure 7d–e). Moreover, we found that overexpression of HUR increased the expression of MAPKAPK3 in U251 and U87 cells with TSPO knockdown (Figure 7f–g). Further, overexpression of HUR increased the mRNA stability of MAPKAPK3 in U251 and U87 cells with low TSPO expression (Figure 7h).

### TSPO regulated the proliferation and mobility depending on the HUR/MAPKAPK3 in glioma

**Figure 8.**
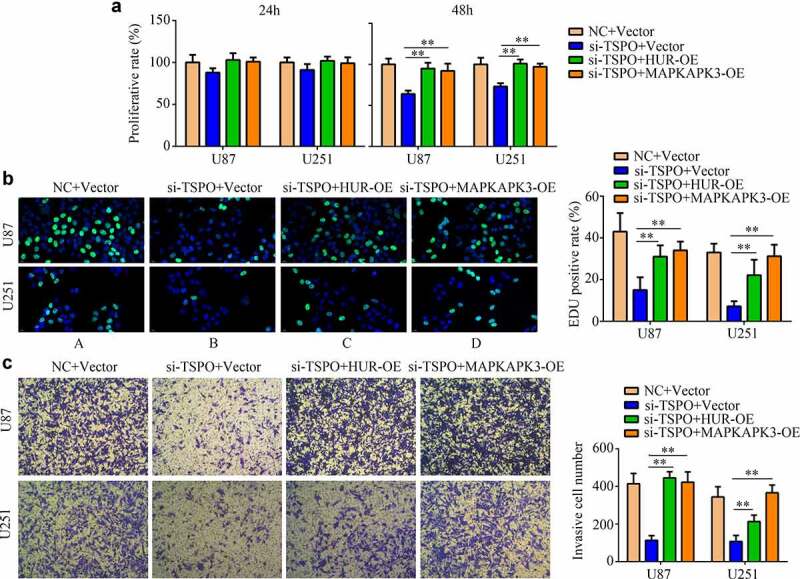
**Overexpression of HUR or MAPKAPK3 reversed the inhibitory effects of TSPO inhibition of glioma cells**. U251 and U87 cells were treated with NC siRNA, TSPO siRNA, TSPO siRNA + HUR-OE, and TSPO siRNA + MAPKAPK3-OE. (a) The CCK-8 assay was used to detect the proliferative rate of U87 and U251 cells in each group treated with the aforementioned conditions in 24 and 48 h. (b) EDU assays were used to detect the EDU-positive rate in each group. (c) Transwell assay was performed to detect the number of invasive cells in each field in each group. * *P* < 0.05; ** *P* < 0.01.

To determine the effects of TSPO in glioma cells via the HUR/MAPKAPK3, rescue experiments were performed. Overexpression of the HUR and MAPKAPK3 significantly relieved the inhibitory effects of TSPO suppression in U87 and U251 cell viability in 48 h (Figure 8a), as well as increased its rapid proliferation in U87 and U251 cells (Figure 8b). Furthermore, overexpression of HUR and MAPKAPK3 in TSPO-inhibited cells increased cell invasion ability (Figure 8c).

## Discussion

Gliomas are the most common primary malignant brain tumors in adults derived from the glial cell lineage [23]. Despite considerable advances in surgical techniques, improvement in radiotherapy approaches, and the arrival of the chemotherapeutic drug temozolomide, the overall survival rates of patients diagnosed with glioma continue to be dismal due to its malignant biological characteristics. Therefore, understanding the underlying molecular mechanisms of gliomas is essential.

Previous clinical research revealed that TSPO expression levels are elevated in various cancers including glioma [24,25], and increased TSPO expression was positively correlated with poor prognosis, as observed in our present study. In fact, the biological function of TSPO in glioma cells had been widely studied. For instance, Rechichi et al. exhibited that overexpression of TSPO in C6 rat glioma cells via the transfecting plasmid increased cell proliferation and migration [26]. Gao et al. demonstrated that the decreased expression of TSPO in glioma cells using paeoniflorin inhibited glioma cell proliferation [27]. In the present study, inhibition of TSPO expression significantly decreased the proliferation and mobility of U251 and U87 cells, as well as the EMT phenotype, in vitro. Interestingly, TSPO knockdown did not significantly affect NHA cell proliferation and mobility. These evidences suggested that TSPO may be distinguished for glioma therapy.

Genes involved in the same biological functions commonly have a co-expression relationship. To determine the effect of TSPO on glioma cell proliferation and mobility, first, the co-expressed genes of TSPO in glioma were analyzed. We found that the co-expressed genes of TSPO were mostly enriched in the MAPK pathway. The MAPK/Ras signaling cascade regulates protein synthesis during neuronal differentiation [28] and is essential for CREB activation [29]. Similarly, activating the MAPK pathway was a pivotal driving force to promote glioma cell proliferation, mobility, and drug resistance [30]. Especially, among members of the MAPK pathway, it was found that MAPKAPK3 had the highest co-expression score. MAPKAPK3 is a member of a family of kinases that acts as an integration point for multiple biochemical signal pathways and affects cellular processes such as proliferation, differentiation, transcription, and development [31]. Accumulating evidence revealed that positive expression of MAPKAPK3 is associated with a poor prognosis in several cancers [32,33]. Then, we determined whether TSPO had the potential to regulate MAPKAPK3. It was demonstrated that inhibition of TSPO decreased the mRNA and protein levels of MAPKAPK3, as well as the activation of its direct downstream CREB and the mRNA stability of MAPKAPK3. Notably, previous studies have indicated the role of TSPO in vascular remodeling during neointima formation through MAPK signaling [34]. The TSPO ligand used to activate TSPO could increase the activation of CREB and improved mood disorder as well as patient memory [35]. The evidence obtained reinforced our results and provided an avenue to uncover the effects of TSPO on CREB activation.

To further investigate the regulatory effect of TSPO on MAPKAPK3 expression, an IP assay of TSPO was carried out and mass spectrometry analysis performed, and it was found that TSPO can bind to many splicing-complex-related proteins, such as HUR, a protein highly expressed in glioma tissue. HUR is normally localized within the nucleus, which can be recruited to the cytoplasm where HUR stabilizes plenty of target mRNAs, many of which encode proteins involved in cell growth, tumorigenesis, angiogenesis, tumor inflammation, invasion, and metastasis [36,37]. Therefore, this study focused on HUR and found that silenced TSPO expression did not affect HUR expression, but promoted nucleus-to-cytoplasm shuttling of HUR. Knockdown of HUR significantly reduced the expression of MAPKAPK3, as well as the proliferation and mobility of glioma cells; overexpression of MAPKAPK3 reversed the effects of HUR knockdown. Moreover, overexpression of HUR reversed TSPO-silencing-induced MAPKAPK3 reduction and mRNA degradation. In addition, overexpression of MAPKAPK3 and HUR could reverse TSPO-silencing-induced inhibition of glioma cell proliferation and mobility.

## Conclusion

Increased TSPO in glioma cells may attract HUR to shuttle from the nucleus to the cytoplasm, promoting HUR binding and increasing the mRNA stability of MAPKAPK3, thus activating the oncoprotein CREB and promoting glioma cell proliferation and mobility.

## Supplementary Material

Supplemental Material

## Data Availability

The datasets used and analyzed in this study are available from the corresponding authors on reasonable request.

## Abbreviations

TSPO, translocator protein 18 kDa; MAPK, mitogen-activated protein kinase; MAPKAPK3, MAPK-activated protein kinase 3; EMT, epithelial to mesenchymal transition; HUR, ELAV1-like RNA-binding protein 1; KD, knockdown; CREB, cAMP-response element-binding protein; LGG, low-grade glioma; GBM, glioblastoma; NHA, normal human astrocytes.

## Highlights

- Inhibition of TSPO reduced MAPKAPK3 expression and its mRNA stability.
- TSPO binds with HUR and promotes its nuclear to cytoplasm.
- Knockdown of HUR inhibited the expression of MAPKAPK3.
- HUR promoted MAPKAPK3 mRNA stability in TSPO-knockdown cells.
- HUR and MAPKAPK3 relieved the effects of TSPO knockdown.
