# Supplementary figures and images for: Inhibition of translocator protein 18 kDa suppressed the progression of glioma via the ELAV-like RNA-binding protein 1/MAPK-activated protein kinase 3 axis
Source: Bioengineered. 2022 Mar 12;13(3):7457–70. doi: 10.1080/21655979.2022.2048992 (PMC9208533; doi:10.1080/21655979.2022.2048992)

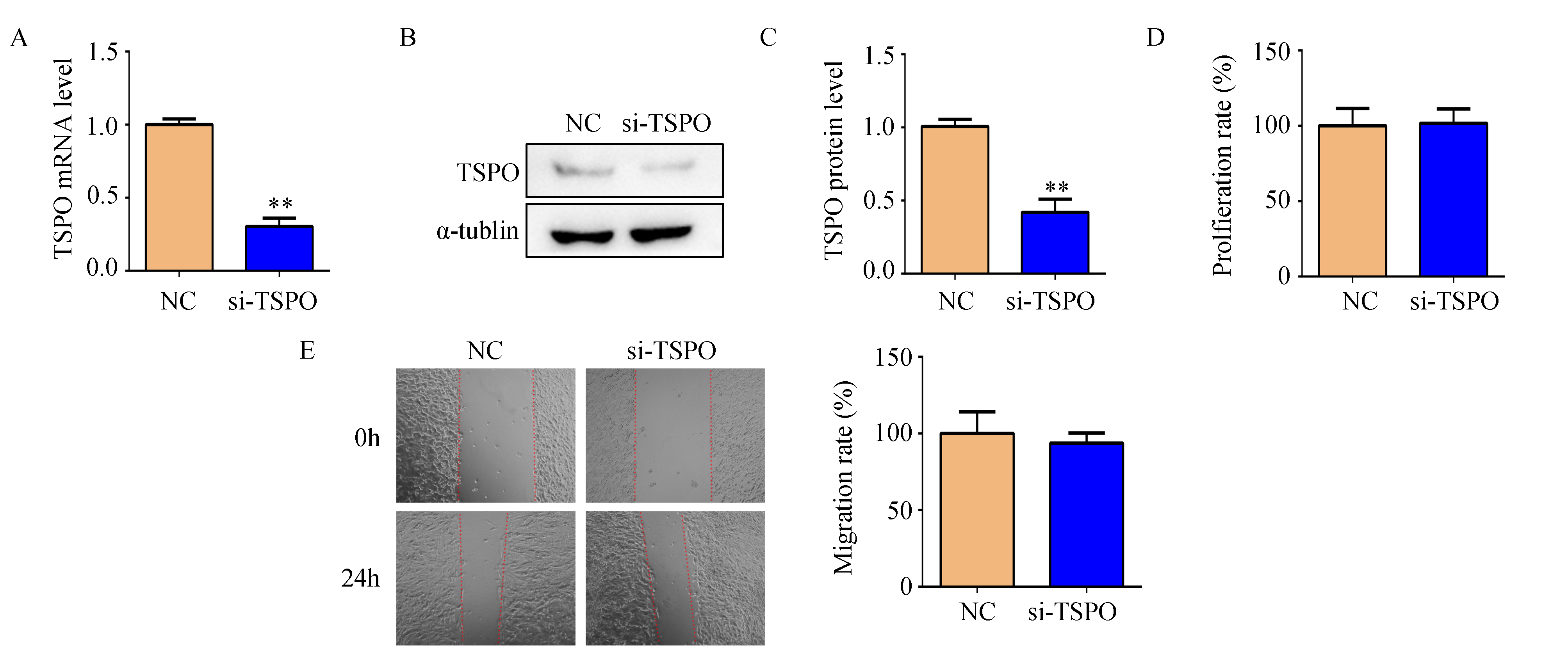

Supplement: Supplemental Material [file KBIE_A_2048992_SM0068.tif]
